# Obituary

**Published:** 1851-05

**Authors:** 


### OBITUARY.

Prof. J. B. Beck, of the College of Physicians and Surgeons of N. Y., died on the 9th ultimo. Dr. Beck was one of the most learned and honored of our profession in this country.

